# Mifepristone induced liver injury in a patient with Cushing syndrome: a case report and review of the literature

**DOI:** 10.1186/s13256-022-03696-x

**Published:** 2023-02-03

**Authors:** Taylor A. Ault, David R. Braxton, Rebecca A. Watson, Alan O. Marcus, Tse-Ling Fong

**Affiliations:** 1 Hoag Digestive Health Institute, Hoag Memorial Hospital Presbyterian, One Hoag Drive, Newport Beach, CA 92663 USA; 2 Department of Pathology, Hoag Memorial Hospital Presbyterian, Newport Beach, CA USA; 3 South Orange County Endocrinology, Laguna Hills, CA USA

**Keywords:** Drug-induced liver injury, Cholestasis, Endothelialitis, Anabolic steroids, Roussell Uclaf Causality Assessment Method

## Abstract

**Background:**

Mifepristone, also known as RU-486, is an anti-progestational steroid with similar chemical structure to anabolic steroids. Given as a single dose in conjunction with misoprostol, mifepristone is used to induce medical abortion. Mifepristone administered chronically at a higher dose is also approved for the management of hypercortisolism. There have been only 2 reported cases of mifepristone associated liver injury, in both cases, in the setting of Cushing syndrome. We report a third patient with Cushing syndrome with mifepristone induced liver injury with unique histological findings that provide insight to the pathophysiology of liver injury in mifepristone and anabolic steroids.

**Case presentation:**

Patient is a 63-year-old Caucasian female Cushing disease with no prior history of liver disease. She was started on mifepristone and semaglutide. Ninety days after initiating mifepristone, she developed deep jaundice, severe pruritus, fatigue, and nausea. Liver tests revealed a mixed hepatocellular/cholestatic pattern. Viral and autoimmune serologies were negative and there was no biliary dilatation on imaging. Liver biopsy showed severe cholestasis but no bile duct injury. Focal endothelialitis was present within a central venule. Cholestatic symptoms persisted for one month after presentation before slowly subsiding. Four months after stopping mifepristone, the patient’s symptoms completely resolved, and liver tests became normal. Compilation of Roussell Uclaf Causality Assessment Method score indicated probable causality.

**Conclusions:**

Mifepristone shares a similar chemical structure as synthetic anabolic/androgenic steroids and there are many similarities in the clinical presentation of liver injury. This case and the 2 other reported cases share similar clinical characteristics. The observation of endothelialitis in our patient may provide a mechanistic link between mifepristone, or anabolic steroids in general, and the development of vascular complications such as peliosis.

## Background

Mifepristone, also known as RU-486, is an anti-progestational steroid administered in a single 600 mg dose in conjunction with misoprostol that is used to induce abortion in up to 10 weeks of gestation [1]. Mifepristone is also approved and marketed as Korlym, prescribed at a dose of 300 mg to 1200 mg daily, for the management of hypercortisolism associated with glycemic abnormalities in patients for whom surgery is not an option [2]. The most common side effects of mifepristone are abdominal/stomach pain, uterine cramping, back pain, diarrhea, dizziness, headache, nausea, and vomiting [3]. The average elimination half-life of mifepristone is 18 hour. Metabolism of mifepristone is primarily via the cytochrome P450 system involving N-demethylation and terminal hydroxylation of the 17-propynyl chain [3, 4].

There have been only 2 reported cases of mifepristone associated liver injury [5, 6]. In both cases, patients were taking mifepristone for the management of Cushing syndrome. The Food and Drug Administration (FDA) Adverse Event Reporting System (FAERS) is a database that comprises serious adverse events, medication error reports and product quality complaints that are submitted to the FDA. There were over 6800 adverse events for mifepristone (Korlym) reported to the FAERS up through early 2022. When the inquiry was limited to liver-related adverse events (using the terms: jaundice, hepatic failure, cholestasis, liver injury, drug-induced liver injury, liver function test increased, liver function test abnormal, liver function test decreased, hepatic function abnormal, hepatic enzyme increase, hepatitis, hepatitis acute, and hepatitis cholestatic) a total of 49 documented cases were identified in the database. Three of these 49 cases (6.1%) cases resulted in deaths. Despite multiple attempts using the Freedom of Information Act, we were unable to obtain information on these cases from the FDA.

We report a third patient with mifepristone induced liver injury and used the updated Roussel Uclaf Causality Assessment Method (RUCAM) to determine causality [7]. The clinical and histology findings of this patient were compared to the 2 prior cases of mifepristone liver injury.

## Case report

**Fig. 1:**
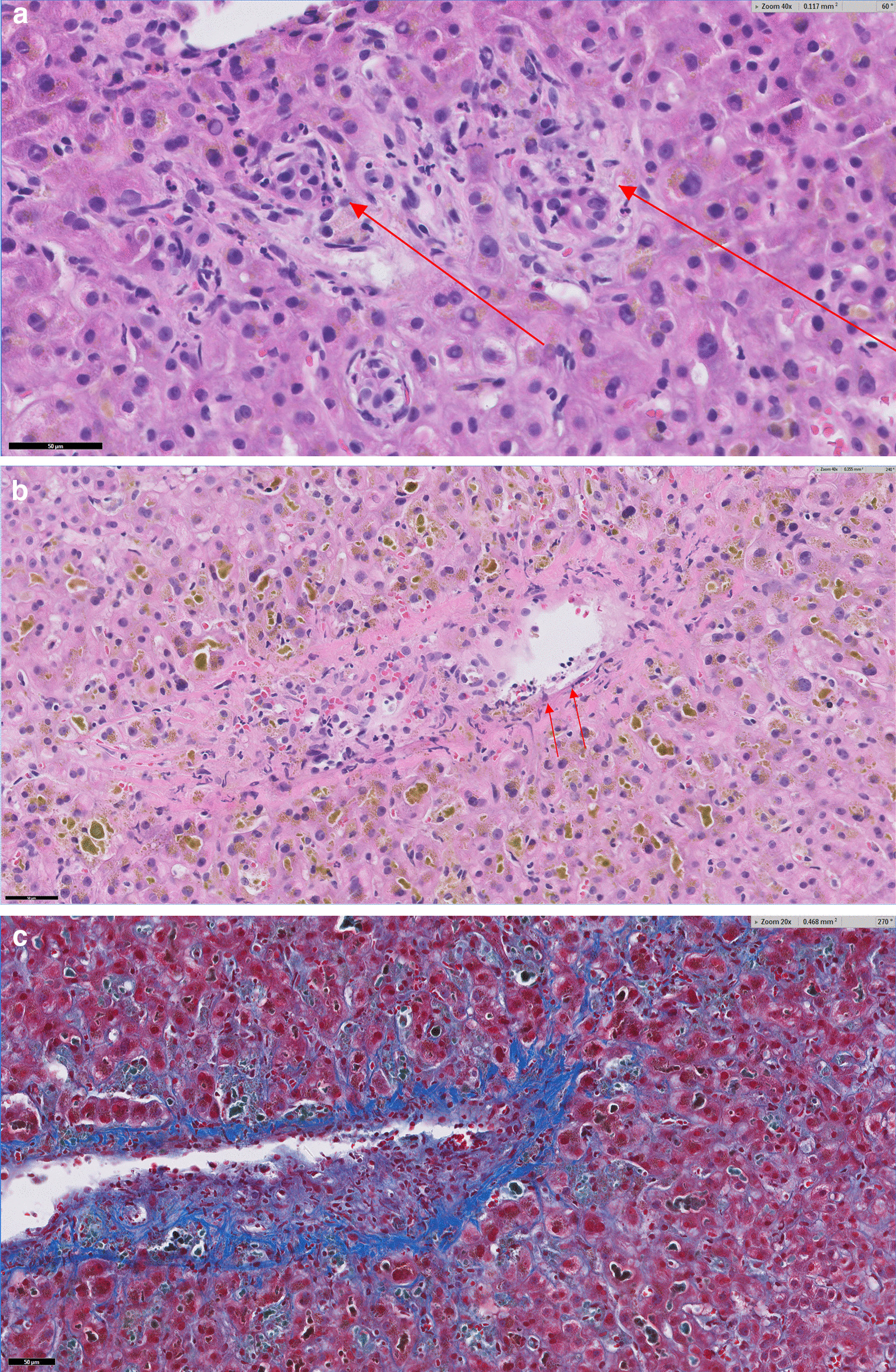
Histopathology of cholestasis with inflammation: **A** The portal tracts are mostly unremarkable but show scattered areas of mild neutrophilic infiltrates (Arrow head) and the lobules show marked cholestasis and rare apoptotic hepatocytes (arrows). **B** shows a rare focus of central venule with endothelialitis (arrows demonstrate lifting of endothelium by inflammatory cells). **C** Trichrome stain reveals mild pericellular fibrosis in the central lobular areas without significant portal fibrosis

Patient is a 63-year-old Caucasian female with history of hypothyroidism who developed insulin resistant diabetes mellitus manifested by 20-pound weight gain and anxiety. She was diagnosed with pituitary microadenoma and Cushing disease. Her baseline liver tests were normal. The patient declined pituitary surgery and she was started on mifepristone 300 mg per os daily and semaglutide 0.5 mg subcutaneous injection weekly. Seven and a half weeks later, the dose of mifepristone was increased to 300 mg twice daily. Alkaline phosphatase level had increased to 187 U/dL but the rest of her liver tests including bilirubin level and aminotransferase activities were initially normal. Five weeks after dose increase of mifepristone, she developed jaundice, fatigue, and nausea. The patient did not have abdominal pain, fever, or chills. Semaglutide and mifepristone were discontinued. The patient drank moderately. Her vital signs were normal, and her physical exam was significant for deep jaundice and the liver edge was palpable at the costal margin. Laboratory studies; alkaline phosphatase 147 U/L, total protein 6.3 g/dL, albumin 4.1 g/dL, total bilirubin 11.3 mg/dL, aspartate aminotransferase (AST) 68 U/L, alanine aminotransferase (ALT) 73U/L, lactate dehydrogenase (LDH) 390 U/L, international normalizing ratio (INR) 1.1, white blood count 8600, 4% eosinophil, hemoglobin 11.8 g/dL, platelet 397,000. Viral (anti-HAV IgM, HBsAg, anti-HBc IgM and anti-HCV) and autoimmune (anti-nuclear antibody, anti-smooth muscle antibody, anti-mitochondrial antibody) serologies were negative, IgA 86 mg/dL, IgM 60 mg/dL, IgG 567 mg/dL, thyroid stimulating hormone 7.69 µIU/mL, free T4 1.0 ng/dL. Magnetic resonance imaging and cholangiography showed liver measuring 17.5 cm, contracted gallbladder with no intra- or extra-hepatic biliary ductal dilatation. Liver biopsy showed cholestasis with inflammation characterized by marked centrilobular canalicular cholestasis, mild neutrophilic infiltrates of the lobules and portal tracts with rare eosinophils and minimal apoptotic hepatocytes (Fig. 1A). There was no significant lymphoplasmacytic infiltrates or steatosis. Importantly, there was no bile duct injury identified within the core biopsy material. Interestingly, focal endothelialitis was present within a central venule (Fig. 1B). Only minimal fibrosis was appreciated in the sinusoidal space by Trichrome stain (Fig. 1C).

**Fig. 2:**
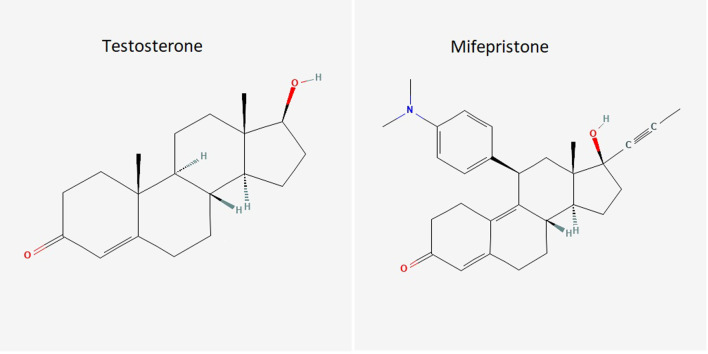
Chemical structure comparison between testosterone and mifepristone

She developed worsening pruritus despite taking ursodeoxycholic acid for 2 weeks. Her cholestatic symptoms persisted for one month before slowly subsiding. (Fig. 2) Four months after stopping mifepristone, the patient’s symptoms completely resolved, and liver tests became normal (Table 1). Rechallenge was not performed. Compilation of Roussell Uclaf Causality Assessment Method (RUCAM) score indicated probable causality [7] (Table 2).

**Table 1:** Liver tests of patient

	Alkaline Phosphatase (U/L)	Total Bilirubin (mg/dL)	Alanine Aminotransferase (U/L)	Aspartate Aminotransferase (U/L)	Comments
Baseline liver tests	82	0.7	21	26	Start mifepristone 300 mg daily
Week 7	187	0.6	15	26	
Week 8					Increase mifepristone 300 mg bid
Week 13	147	11.3	73	68	Mifepristone discontinued
Week 14	178	23.0	46	76	Start Ursodiol 300 mg daily
Week 15	234	28.3	38	76	Peak bilirubin
Week 19	299	7.1	192	172	Peak alkaline phosphatase and aminotransferase activities
Week 21	233	3.6	142	103	
Week 25	124	1.2	71	52	
Week 35	105	0.5	36	36	
Week 40	84	0.4	30	36	Complete resolution

**Table 2:** RUCAM for liver injury

	Possible score	Study patient
Time to onset		
5–90 days	+ 2	+ 2
< 5 or > 90 days	+ 1	
Course of alkaline phosphatase after cessation of drug		
Decrease > 50% within 8 days	+ 3	0
Decrease > 50% within 30 days	+ 2	
Risk factors		
Alcohol +	− 1	
Alcohol −	0	0
Age ≥ 55 years	+ 1	+ 1
Age < 55 years	0	
No concomitant drugs/herbs		0
All causes-group I and II-ruled out		+ 2
Previous hepatotoxicity		
Reaction labeled in the product characteristics	+ 2	+ 1
Reaction published but not labeled	+ 1	
Response to unintentional re-exposure		0
Total score for the case		+ 6

## Discussion

We are reporting a third case of drug induced liver injury (DILI) caused by mifepristone. Like the 2 previous cases, mifepristone was prescribed for the treatment of Cushing disease. Using the updated RUCAM, the score indicated probable causality [7] (Table 2). There are no reported cases of liver injury where mifepristone was taken as an emergency contraception. Our patient was also taking semaglutide, a glucagon-like peptide inhibitor, which has been associated with an increased risk of cholelithiasis, cholecystitis and biliary disease when used in longer duration and for weight loss [8]. However, there has not been a single reported case of DILI associated with semaglutide [4] although its role in this case cannot be entirely excluded.

The clinical course and pattern of liver injury of this patient and the 2 previously reported cases are similar [5, 6] (Table 3). All 3 patients were women, and the latency period was 90 days. Our patient developed increasing alkaline phosphatase prior to the onset of jaundice. These patients all experienced deep jaundice (bilirubin > 20 mg/dL) and pruritus. The R factor was low (mixed cholestatic hepatocellular liver injury) but was < 2 in only one case (cholestatic liver injury) [9]. Prothrombin time was intact and none of the patients had mental status changes. All patients recovered, but the time from presentation to recovery was protracted; lasting 3 months for symptoms resolution, and 40 weeks for normalization of liver tests.

**Table 3:** Summary of demographic and laboratory of patients with mifepristone-induced liver injury

Patient characteristics	Patient in this case report	Ref Funke [5]	Ref Shah [6]
Age	63	65	35
Gender	Female	Female	Female
Ethnicity	Caucasian	Caucasian	Not available
Time to onset (days)	90	90	90
Peak bilirubin (mg/dL)	28.3	33	21.4
Peak alkaline phosphatase (U/L)	299	188	258
Peak ALT (U/L)	231	169	68
Peak AST (U/L)	189	53	56
Presentation to peak bilirubin (weeks)	3	8	Not available
Presentation to peak alkaline phosphatase (weeks)	8	8	Not available
Presentation to peak ALT (weeks)	8	8	Not available
Peak INR	1.2	1.44	“within normal limits”
R factor	2.3	2.7	1.6
Time from peak to resolution (weeks)	40	40	

*ALT* alanine aminotransferase, *AST* aspartate aminotransferase; *INR* international normalizing ratio

The histology of this case differs somewhat from previous reports. The reports by Funke *et al.* [5], and Shah *et al.* [6], describe a bland cholestasis, which is typically defined as cholestasis without significant necro-inflammatory activity. In contrast, the liver histology of our patient shows cholestatic hepatitis with significant neutrophilic and eosinophilic inflammation that accompanies the cholestasis along with rare apoptotic hepatocytes and a focus of central venular endothelialitis. The liver histology is consistent with the biochemical pattern of liver injury of this patient. Mifepristone, also known as 11β-(4-(dimethylamino)phenyl)-17α-(1-propynyl)estra-4,9-dien-17β-ol-3-one, is a synthetic estrange steroid. In having a 17-Carbon ring structure, mifepristone shares this fundamental chemical structure similar to synthetic anabolic–androgenic steroid. In addition to having a phenyl-amino-dimethyl group on Carbon 11, mifepristone has a propynyl group on Carbon 17, making it a C-17α alkylated steroid [10] (Fig. 2).

As previously noted [3], the clinical picture of mifepristone liver injury is almost identical to cholestasis caused by anabolic/androgenic steroids with respect to latency, biochemical pattern of liver injury and histology. Duration of cholestasis observed in the 3 cases of mifepristone liver injury is also similar to the protracted course in anabolic/androgenic steroids [11–15] Endothelialitis is typically associated with liver allograft rejection, immune checkpoint inhibitor mediated injuries, viral hepatitis, COVID-19 infection, and radiation hepatitis [16, 17]. However, anabolic steroid use has been traditionally associated with the complication of peliosis hepatis, a condition in which the endothelial lining of the sinusoids is lost and large blood-filled lakes form masses within the hepatic parenchyma [18]. The observation of endothelialitis in our patient may provide a mechanistic link between mifepristone, or anabolic steroids in general, and the development of vascular complications such as peliosis.

## Conclusion

In summary, we report the third case of cholestatic liver injury associated with mifepristone prescribed for the management of Cushing syndrome. This case and the 2 other reported cases share similar characteristics; 90-day latency, deep jaundice, and a protracted recovery. Additional cases of mifepristone cases have been reported in the FDA MedWatch database. Mifepristone shares a similar chemical structure as synthetic anabolic/androgenic steroids and there are many similarities in the clinical presentation of liver injury. The observation of endothelialitis on the liver biopsy of our patient may provide a mechanistic link between mifepristone, or anabolic steroids in general, and the development of vascular complications such as peliosis.

## Data Availability

The datasets during and/or analyzed during the current study available from the corresponding author on reasonable request.
